# Health resource utilization associated with skeletal-related events in patients with advanced breast cancer: results from a prospective, multinational observational study

**DOI:** 10.1186/2193-1801-3-328

**Published:** 2014-06-30

**Authors:** Diana Lüftner, Vito Lorusso, Ignacio Duran, Guy Hechmati, Cristina Garzon-Rodriguez, John Ashcroft, Amit Bahl, Prayashi Ghelani, Rachel Wei, Emma Thomas, Herbert Hoefeler

**Affiliations:** Charité Universitätsmedizin Berlin, Berlin, Germany; National Cancer Institute IRCCS Giovanni Paolo II, Bari, Italy; Oncology Institute ASL, Lecce, Italy; Centro Integral Oncológico Clara Campal (CIOCC), Madrid, Spain; Health Economics, Amgen (Europe) GmbH, Zug, Switzerland; Instituto Catalán d’Oncología ICO-IDIBELL, Barcelona, Spain; Pinderfields General Hospital, Wakefield, UK; University Hospitals Bristol, Bristol, UK; Biostatistics, Ovatech Solutions, London, UK; Biostatistics, Amgen Inc, Thousand Oaks, CA USA; Scientific Publications, Amgen (Europe) GmbH, Zug, Switzerland; Forschungszentrum Ruhr, Witten, Germany

**Keywords:** Bone metastases, Breast cancer, Europe, Health resource utilization, Skeletal-related events

## Abstract

Patients with breast cancer and bone metastases often experience skeletal complications (skeletal-related events [SREs]: pathologic fracture, radiation to bone, surgery to bone or spinal cord compression). Prospective data on the health resource burden of SREs are needed for planning healthcare requirements and estimating the value of new treatments, but limited data are available. This prospective, observational study collected health resource utilization (HRU) data independently attributed to SREs by investigators. Eligible patients had bone metastases secondary to breast cancer, life expectancy ≥6 months, Eastern Cooperative Oncology Group (ECOG) performance status ≤2, and at least one SRE in the 97 days before enrollment. Data, collected retrospectively for 97 days before enrollment and prospectively for 18–21 months, included number and duration of inpatient stays, outpatient visits, emergency room visits and procedures. Altogether, 223 patients were enrolled from Germany, Italy, Spain and the UK. Of the 457 SREs, 118 (25.8%) were associated with inpatient stays. The mean duration of stay was 19.5 (standard deviation [SD] 19.2) days per SRE (based on 117 SREs). Surgery to bone and spinal cord compression were the SREs most likely to require inpatient stays (77.8% and 57.9% of SREs, respectively), while radiation to bone was the least likely (9.7%). Spinal cord compression required the longest inpatient stay per event (34.2 [SD 30.2] days) and radiation to bone the shortest (14.3 [SD 10.2] days). Overall, 342 SREs (74.8%) required an outpatient visit, with radiation to bone the most likely (85.7%), and surgery to bone the least likely (42.6%). Radiation to bone was also associated with the greatest number of outpatient visits per event (6.8 [SD 6.7] visits). All SREs were associated with substantial HRU therefore, preventing SREs in patients with breast cancer may reduce the burden imposed on healthcare systems.

## Introduction

Breast cancer is the most common cancer in women, with nearly half a million new cases diagnosed in Europe in 2008 (Steliarova-Foucher et al. 2012). Up to 75% of patients with advanced breast cancer develop bone metastases (Coleman 1997), which are often associated with skeletal or bone complications, otherwise referred to as skeletal-related events (SREs) and commonly comprising pathologic fracture, radiation to bone, surgery to bone, and spinal cord compression (Coleman 2001). The high frequency of bone metastases and associated complications in patients with breast cancer means SREs contribute significantly to the clinical and economic burden of the disease.

SREs can be debilitating and patients frequently experience a reduction in quality of life, with significant decreases in physical and functional well-being (Weinfurt et al. 2004). SREs are associated with considerable morbidity, the worst being spinal cord compression, which can lead to paralysis, and pathologic fractures, which may result in disability or the need for surgery, with the potential for additional perioperative morbidity (Coleman 2006; Katzer et al. 2002). As might be expected, SREs often result in considerable pain, which further impairs patient mobility and reduces quality of life (Katzer et al. 2002; DePuy et al. 2007; Costa et al. 2008). SREs are also associated with an increased risk of death (Norgaard et al. 2010; Saad et al. 2007).

In addition to the patient impact, SREs impose a substantial burden on healthcare resources. Planning future resource requirements and estimating the value of new treatment options requires data on the resource burden of SREs. However, there is a lack of prospective data on the impact of SREs on health resource utilization (HRU).

Several retrospective US studies have suggested that SREs increase HRU (Delea et al. 2004, 2006; Lage et al. 2008; Schulman and Kohles 2007), and studies in Spain, France, and Portugal have demonstrated that SREs increase HRU and costs (Pockett et al. 2010; Decroisette et al. 2011; Felix et al. 2011; Svendsen et al. 2013). However, these studies were restricted to individual countries and to our knowledge there have been no large, international, prospective studies investigating the differential contribution of the various types of SREs to HRU.

We therefore designed a prospective, observational, multinational study to evaluate the HRU associated with each type of SRE in patients with bone metastases or lesions secondary to breast, prostate or lung cancer, or multiple myeloma in Canada, Germany, Italy, Spain, the United Kingdom (UK) and the United States of America (USA). Here, we report the data for patients with advanced breast cancer from the four European countries (Germany, Italy, Spain, and the UK).

## Methods

### Patients

Patients aged 18 years or older with bone metastases secondary to breast cancer and a life expectancy of at least 6 months were eligible for inclusion in the study. In addition, patients were required to have an Eastern Cooperative Oncology Group (ECOG) performance status of 0, 1 or 2, and to have experienced at least one SRE in the 97 days before signing informed consent or up to 7 days afterward. Patients who were enrolled in an investigational drug trial for treatment of bone metastases or prevention of SREs were excluded from the study.

### Study design

Patient demographics and information on disease history were collected at enrollment. HRU data for each patient were collected retrospectively by chart review for all SREs occurring in the 97-day period before enrollment, and prospectively for the duration of their involvement in the study. The planned follow-up period for patients in the study was up to 18–21 months. SREs were defined as pathologic fracture (either vertebral or non-vertebral), radiation to bone, surgery to bone, or spinal cord compression; investigators independently attributed HRU to SREs. HRU outcome measures included: the number, duration and facility type of inpatient stays; the number and facility type of outpatient visits; the number of emergency room visits; the number, duration and facility type of nursing home/long-term care facility stays; the number of home health visits; and the type of procedure.

### Statistical analyses

As described previously (Hechmati et al. 2011), all analyses were descriptive. HRU was summarized by SRE type, and the mean HRU per SRE type was calculated by dividing the total HRU attributed to SREs by the total number of SREs of the same type. The mean duration of inpatient stay per SRE was calculated as the total number of inpatient days divided by the total number of SREs that were associated with an inpatient stay (if an SRE resulted in multiple inpatient stays, the total duration of all inpatient stays was used). Data on inpatient stays by facility type reported SREs with at least one inpatient stay within the facility type and SRE type. When an SRE required stays in more than one facility type, a stay was attributed to each facility type.

If an SRE required multiple inpatient stays within one facility type, the total duration of inpatient stays was counted. If radiation or surgery to bone was carried out as a result of another SRE (i.e. treatment of a primary SRE, such as pathologic fracture), the investigator had the option of attributing HRU to the primary SRE. Therefore, SREs determined to be secondary to a primary SRE were excluded from the analysis.

The data are primarily reported as mean values, rather than medians, because this better describes the total resources used at a population level: information that is required for healthcare policy decisions (Thompson and Barber 2000). Median values are also reported in the figures to illustrate the distribution of data when sample sizes are small, and to describe the typical HRU for an individual patient.

## Results

### Study population

At the time of the final analysis, 223 patients with a primary diagnosis of breast cancer who met the eligibility criteria were enrolled across the European sites. The mean (standard deviation [SD]) length of follow-up for these patients ranged from 7.1 (5.2) to 10.3 (5.4) months (Table 1).

**Table 1:** **Baseline demographics and disease history**

Characteristic	Germany (***n*** = 85)	Italy (***n*** = 62)	Spain (***n*** = 31)	UK (***n*** = 45)
Follow-up time, months, mean (SD)	10.3 (5.4)	8.6 (4.8)	7.1 (5.2)	7.4 (5.5)
Female, *n* (%)	84 (98.8)	61 (98.4)	28 (90.3)	45 (100.0)
Ethnic group, *n* (%)				
White or Caucasian	85 (100.0)	61 (98.4)	30 (96.8)	44 (97.8)
Other^a^	0 (0.0)	1 (1.6)	1 (3.2)	1 (2.2)
Age, years, median (range)	62.0 (40, 81)	60.0 (36, 83)	56.0 (38, 79)	58.0 (32, 92)
≥ 65 years, *n* (%)	34 (40.0)	21 (33.9)	11 (35.5)	17 (37.8)
ECOG performance status, *n* (%)				
0	27 (31.8)	18 (29.0)	6 (19.4)	14 (31.1)
1	41 (48.2)	31 (50.0)	12 (38.7)	21 (46.7)
2	17 (20.0)	13 (21.0)	13 (41.9)	10 (22.2)
History of SREs^b^ *n* (%)	53 (62.4)	34 (54.8)	20 (64.5)	21 (46.7)
Time since primary cancer diagnosis, months, median (Q1, Q3)	70.8 (22.9, 132.2)	39.0 (8.7, 99.1)	62.7 (27.5, 138.4)	72.2 (33.3, 118.0)
Time since bone metastasis diagnosis, months, median (Q1, Q3)	4.4 (1.8, 22.9)	4.1 (1.8, 13.3)	4.9 (1.8, 39.6)	9.6 (3.0, 32.2)

^a^‘Other’ includes Asian and Hispanic or Latino ethnic groups; ^b^before the 90-day period preceding the signing of informed consent.

ECOG-Eastern Cooperative Oncology Group, Q-quartile, SD-standard deviation, SRE-skeletal-related event, UK-United Kingdom.

Baseline characteristics and disease history were generally similar across the four countries (Table 1). There was a higher proportion of patients with an ECOG performance status of 2 in Spain (41.9%) than in the other countries (20.0–22.2%), and a correspondingly lower proportion of patients with ECOG performance status of 0 (19.4% vs. 29.0–31.8%) and 1 (38.7% vs. 46.7–50.0%). The median time from diagnosis of the primary cancer to enrollment was notably shorter for patients in Italy (39.0 months) than for the other countries (62.7–72.2 months), while the median time from bone metastases detection to enrollment was notably longer in the UK (9.6 months) than elsewhere (4.1–4.9 months).

### Skeletal-related events

**Figure 1:**
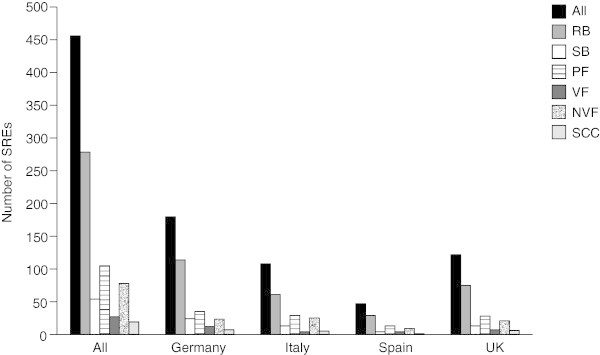
**Number of SREs included in the HRU analysis by country and by SRE type.** VF and NVF are subsets of PF. *NVF* non-vertebral fracture, *PF* pathologic fracture, *RB* radiation to bone, *SB* surgery to bone, *SCC* spinal cord compression, *SRE* skeletal-related event, *VF* vertebral fracture.

Eligible patients experienced a total of 489 SREs. Analysis of the crude SRE data (including SREs excluded from the HRU analysis) showed that the SRE rate per patient-year was consistent across Germany, Italy, and Spain (2.0 SREs per patient-year), but was higher in the UK (3.3 SREs per patient-year). After excluding any SREs determined by investigators to be secondary to a previous SRE (32 SREs), 457 SREs remained eligible for inclusion in the HRU analysis (Figure 1). Radiation to bone was the most common SRE, accounting for 279 of the 457 events, while spinal cord compression was the least common (19 events); this pattern was consistent across all countries studied.

### Health resource utilization

#### Inpatient stays

**Figure 2:**
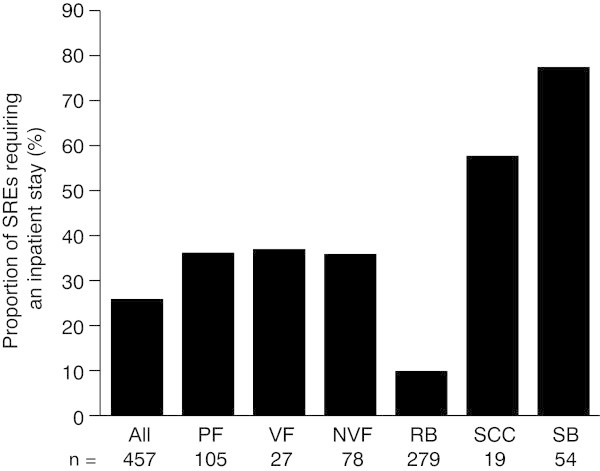
**Proportion of SREs requiring an inpatient stay.** VF and NVF are subsets of PF *n* = number of SREs. *NVF* non-vertebral fracture, *PF* pathologic fracture, *RB* radiation to bone, *SB* surgery to bone, *SCC* spinal cord compression, *SRE* skeletal-related event, *VF* vertebral fracture.

Overall, 118 of 457 SREs (25.8%) required hospitalization. Although all types of SRE contributed to inpatient stays, the proportion of SREs requiring an inpatient stay varied greatly across SRE types (Figure 2). Spinal cord compression and surgery to bone were most likely to require inpatient stays (with 57.9% and 77.8% requiring stays, respectively), while radiation to bone was the least likely (9.7%). The rates of inpatient stays were generally consistent across all countries, with the exception of vertebral fracture; for this SRE, patients in the UK were less likely to be hospitalized (14.3%) than those from other countries (41.7–50.0%), although the overall number of vertebral fractures was low (*n* = 27).

**Figure 3:**
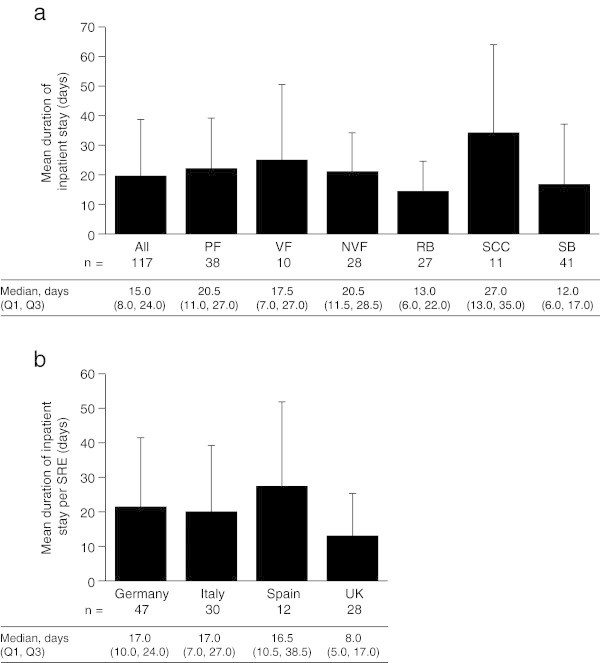
**Mean duration of inpatient stay per SRE that required an inpatient stay. (a)** by SRE type, and **(b)** by country. Data are shown as mean (+standard deviation). Median (Q1, Q3) data are displayed below the graph. Data include only SREs requiring an inpatient stay. If an SRE resulted in multiple inpatient stays, the total duration of all the inpatient stays was used. VF and NVF are subsets of PF. *n* = number of SREs requiring an inpatient stay with at least one inpatient stay by SRE type. *NVF* non-vertebral fracture, *PF* pathologic fracture, *Q* quartile, *RB* radiation to bone, *SB* surgery to bone, *SCC* spinal cord compression, *SRE* skeletal-related event, *UK* United Kingdom, *VF* vertebral fracture.

Data on the duration of inpatient stays were collected for 117 of the 118 SREs that required hospitalization (data for one surgery to bone event were not collected). All types of SREs were associated with inpatient stays of substantial duration (Figure 3a). The mean (SD) length of inpatient stay per SRE that required an inpatient stay was 19.5 (19.2) days. Spinal cord compression required the longest stay per event (34.2 [SD 30.2] days) and radiation to bone the shortest (14.3 [SD 10.2] days). Across the four countries, the overall mean length of stay ranged from 12.9 to 27.2 days, with the UK having the shortest mean duration and Spain the longest (Figure 3b). In Spain, the mean length of stay for pathologic fracture was over twice as long as that for any of the other countries (46.4 [SD 28.5] days vs. 17.2–19.8 days). However, this probably reflects the small number of patients with pathologic fractures requiring inpatient stays who were recruited in Spain (*n* = 5) and the long duration of stay needed for one of the recorded events (87 days). In the UK, the mean length of stay for radiation to bone (6.5 [SD 6.2] days; six events) was less than half that of the overall mean for radiation to bone across all four countries. Data on facility type indicated that the mean length of stay in oncology units/wards (the only contributor to inpatient stays for radiation to bone in the UK) was only 6.5 (SD 6.2) days for the UK, whereas the overall mean for all countries for oncology unit/ward stays for radiation to bone was 11.0 (SD 8.3) days (Table 2).

**Table 2:** **Number and mean duration of inpatient stays (in days) by facility type**

	All		Pathologic fracture		Vertebral fracture ^a^		Non-vertebral fracture ^a^		Radiation to bone		Spinal cord compression		Surgery to bone	
Facility	***n***	Mean (SD)	***n***	Mean (SD)	***n***	Mean (SD)	***n***	Mean (SD)	***n***	Mean (SD)	***n***	Mean (SD)	***n***	Mean (SD)
Surgical unit/ward	50	15.3 (9.2)	18	20.2 (9.2)	1	24.0 (–)	17	19.9 (9.4)	0	–	2	17.0 (5.7)	30	12.2 (8.3)
Oncology unit/ward	39	14.4 (17.7)	7	9.6 (5.1)	3	8.0 (4.6)	4	10.8 (5.8)	20	11.0 (8.3)	8	28.6 (33.4)	4	11.3 (13.9)
Other	18	19.1 (23.5)	8	14.4 (8.8)	2	8.0 (5.7)	6	16.5 (8.9)	2	27.0 (0.0)	0	-	8	21.9 (34.9)
General unit/ward	10	19.9 (25.9)	5	29.0 (35.0)	1	87.0 (–)	4	14.5 (15.4)	4	9.0 (8.7)	1	18.0 (–)	0	–
Radiation unit/ward	8	20.8 (12.7)	2	24.5 (3.5)	2	24.5 (3.5)	0	–	4	18.8 (18.0)	2	21.0 (9.9)	0	–
Rehabilitation unit/ward	6	29.8 (10.8)	2	36.0 (18.4)	1	49.0 (–)	1	23.0 (–)	0	–	1	22.0 (–)	3	28.3 (7.1)
Gynecology unit/ward	1	33.0 (–)	0	–	0	–	0	–	0	–	1	33.0 (–)	0	–
Intensive care unit/ward	1	4.0 (–)	0	–	0	–	0	–	0	–	0	–	1	4.0 (–)
Nursing facility	1	26.0 (–)	1	26.0 (–)	0	–	1	26.0 (–)	0	–	0	–	0	–
Rehabilitation facility	1	5.0 (–)	0	–	0	–	0	–	0	–	0	–	1	5.0 (–)

^a^Vertebral fracture and non-vertebral fracture are subsets of pathologic fracture.

*n* = Number of SREs with at least one inpatient stay within facility type and SRE type. (If an SRE required stays in more than one facility type, a stay was attributed to each facility type for the appropriate duration. If an SRE had multiple inpatient stays within one facility type, the total duration of inpatient stays was counted).

SD-standard deviation, SRE-skeletal-related event.

Across all SREs, the most common facility types for inpatient stays were surgical units/wards and oncology units/wards, with general units/wards, rehabilitation units/wards, radiation units/wards and ‘other’ units/wards also used frequently (Table 2). Spinal cord compression and radiation to bone were both most likely to be treated in an oncology unit/ward. Patients with pathologic fracture were most likely to stay in a surgical unit/ward, with a mean length of stay of 20.2 (SD 9.2) days. Patients requiring surgery to bone were also most likely to be treated in a surgical unit/ward, but the mean length of stay (12.2 [SD 8.3] days) was lower than average for this type of facility.

#### Outpatient visits

**Figure 4:**
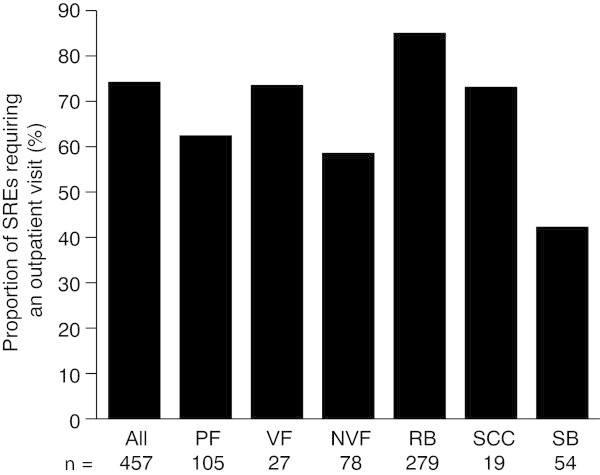
**Proportion of SREs requiring an outpatient visit.** VF and NVF are subsets of PF *n* = number of SREs. *NVF* non-vertebral fracture, *PF* pathologic fracture, *RB* radiation to bone, *SB* surgery to bone, *SCC* spinal cord compression, *SRE* skeletal-related event, *VF* vertebral fracture.

**Figure 5:**
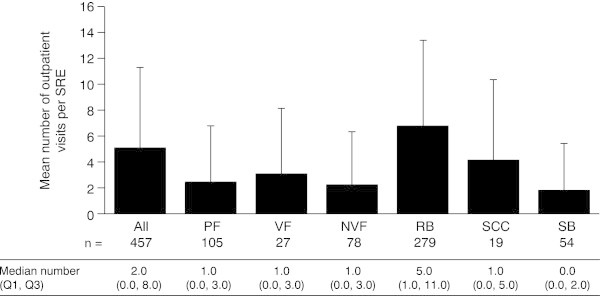
**Mean number of outpatient visits per SRE.** Data are shown as mean (+standard deviation). Median (Q1, Q3) data are displayed below the graph. VF and NVF are subsets of PF. *n* = number of SREs requiring an inpatient stay with at least one inpatient stay within each SRE type. *NVF* non-vertebral fracture, *PF* pathologic fracture, *Q* quartile, *RB* radiation to bone, *SB* surgery to bone, *SCC* spinal cord compression, *SRE* skeletal-related event, *VF* vertebral fracture.

Outpatient visits were also common across all SREs (Figure 4). Overall, radiation to bone required the highest proportion of visits, with 85.7% of events requiring a visit, and surgery to bone the lowest (42.6%). In Spain, however, pathologic fracture was the SRE most likely to require an outpatient visit (76.9%), with a lower proportion of radiation to bone events (72.4%) requiring a visit. The proportion of outpatient visits for surgery to bone showed the most variation across countries, with a visit most likely in the UK (61.5%) and least likely in Spain (25.0%).The mean number of outpatient visits per SRE across all SREs and countries was 5.1 (SD 6.3) (Figure 5). Radiation to bone was associated with the highest number of outpatient visits per SRE (6.8 [SD 6.7] visits), with the number of visits required for other SREs ranging from 1.8 (SD 3.6) for surgery to bone to 4.1 (SD 6.3) for spinal cord compression. In the UK, the mean number of outpatient visits required per SRE (2.5 [SD 2.7]) was half that of the overall mean. The low number of visits required for radiation to bone (2.6 [SD 2.4]) was probably the greatest contributor to this. In Italy, the mean number of visits required for spinal cord compression was low (1.6 [SD 2.5]).

#### Procedures performed

**Figure 6:**
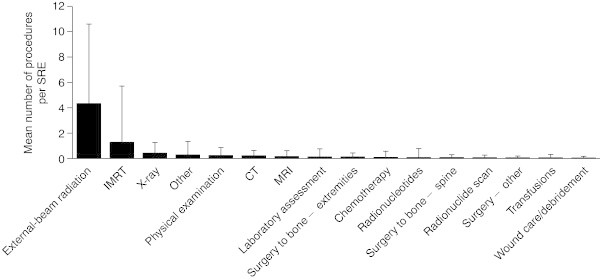
**Mean number of procedures performed per SRE by type.** Median (Q1, Q3) for external-beam radiation: 1.0 (0.0, 8.0); X-ray: 0.0 (0.0, 1.0); all other procedure types: 0.0 (0.0, 0.0). Data are shown as mean (+standard deviation). *CT* computed tomography, *IMRT* intensity-modulated radiation therapy, *MRI* magnetic resonance imaging, *Q* quartile.

**Figure 7:**
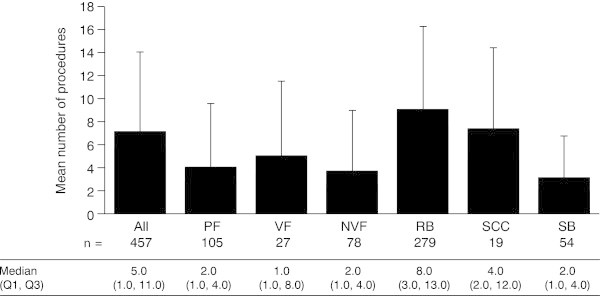
**Mean number of procedures per SRE.** Data are shown as mean (+standard deviation). Median (Q1, Q3) data are displayed below the graph. VF and NVF are subsets of PF. *n* = number of SREs. *NVF* non-vertebral fracture, *PF* pathologic fracture, *Q* quartile, *RB* radiation to bone, *SB* surgery to bone, *SCC* spinal cord compression, *VF* vertebral fracture.

Across all the countries, almost all SREs required a procedure (90.7–100% across the different SRE types), with external-beam radiation the most common procedure type (Figure 6).SREs required a mean of 7.1 (SD 7.1) procedures per SRE overall (Figure 7). Radiation to bone and spinal cord compression were associated with the highest number of procedures (9.1 [SD 7.3] and 7.4 [SD 7.2], respectively), with external-beam radiation the most common procedure performed for both. Most procedures were performed in an outpatient setting (5.4 [SD 6.7] procedures per SRE); 1.5 (SD 4.1) procedures per SRE required an overnight stay, with spinal cord compression most likely to require this. Procedures requiring an emergency room visit were rare (0.1 [SD 0.4] procedures per SRE).

Patients in the UK required the lowest number of procedures for radiation to bone, reflecting the pattern seen for the number of outpatient visits required for this SRE. Notably, the mean rates of external-beam radiation and intensity-modulated radiation therapy (IMRT) per SRE in the UK were low, at 2.0 (SD 2.0) and 0.0, respectively. Germany recorded the highest number of procedures for radiation to bone, which were mostly performed in an outpatient setting. These were mainly external-beam radiation (8.6 [SD 8.2] per SRE) and IMRT (4.0 [SD 7.6] per SRE).

#### Emergency room and home health visits, and nursing home/long-term care facility stays

Very few home health visits (one in every 100 SREs) and emergency room visits (five in every 100 SREs) were reported, and only four patients required a stay in a nursing home/long-term care facility.

## Discussion

This is the first large, prospective study investigating HRU associated with different types of SRE in patients with breast cancer metastatic to bone in Europe. We found that all SREs were associated with substantial HRU, with lengthy hospitalizations frequently required. This is also the first study in which HRU has been assigned to specific SREs independently by the investigators. This ensures that HRU was recorded only when it was deemed by investigators’ expert opinion to be a direct consequence of the SRE, rather than owing to the underlying disease.

Patient characteristics at enrollment were broadly similar across the different countries. However, patients in the UK had a higher rate of SREs per patient-year than those from the three other European countries studied, which may reflect the longer median time from the detection of bone metastases to enrollment in the study in the UK (approximately double that seen for the other countries). Despite this, the ECOG performance status of patients in the UK showed a similar distribution to those from Germany and Italy, whereas Spain had a higher proportion of patients with a poorer ECOG performance status of 2.

In general, patterns of HRU were similar across the four European countries studied. The overall patterns of HRU observed in our study are similar to those reported in studies by Gunther et al. in Austria, the Czech Republic, Poland, Sweden, and Switzerland (Gunther et al. 2011), by Felix et al. in Portugal (Felix et al. 2011), and by Svendsen et al. in Denmark (Svendsen et al. 2013). All of these studies found SREs were associated with lengthy inpatient stays, which were major contributors to the overall cost of care.

Inpatient stays were particularly common with spinal cord compression and surgery to bone. Although spinal cord compression was rare, it required the longest hospital stays. This is consistent with two separate economic analyses illustrating the high cost of each spinal cord compression event (Felix et al. 2011; Barlev et al. 2010). Notably, surgery to bone required shorter inpatient stays than pathologic fracture, probably because surgery facilitates faster stabilization of bone. Radiation to bone is one of the most common SREs in patients with advanced breast cancer (Lipton et al. 2000). Despite requiring inpatient stays less frequently, this SRE was still associated with a considerable proportion of hospitalizations. This may have been owing to complications requiring an inpatient stay or patients being required to travel substantial distances for multifractionated radiotherapy.

Approximately three-quarters of SREs were associated with outpatient visits, with a mean of five visits per SRE. Therefore, outpatient visits may also impose a considerable burden on healthcare resources in Europe. In an analysis of the economic burden of bone metastases in the USA, outpatient visits were found to be the biggest contributor to incremental costs associated with bone metastases, accounting for 63–71% of the additional costs compared with patients without bone metastases (Schulman and Kohles 2007).

Radiation to bone was associated with the greatest number of procedures per SRE, in addition to the highest number of outpatient visits. Our findings therefore suggest that radiation to bone is a major contributor to the increased burden that SREs place on healthcare systems, consistent with similar results in other studies (Lage et al. 2008; Decroisette et al. 2011; Felix et al. 2011). The high numbers of outpatient visits and procedures observed in our study probably reflect the use of multiple fractions of radiotherapy. A study in patients with breast or prostate cancer with bone metastases in Portugal reported a mean of 8.4 sessions per treatment for radiation to bone (Felix et al. 2011). In a retrospective study of palliative radiotherapy for bone metastases in Spain, only 25% of cases received single-fraction (800 cGy) treatment, with 59% receiving five- or ten-fraction regimens (Expósito et al. 2012). The lower number of procedures and outpatient visits reported in the UK for radiation to bone is probably owing to a preference for single-fraction radiotherapy over multiple-fraction treatment. Surveys of radiotherapy for bone metastases in the UK reported a mean of about three fractions per treatment, with approximately 60% of treatments delivered as a single fraction (Williams et al. 2007; Royal College of Radiologists 2007). The length of inpatient stay for radiation to bone was also much shorter in the UK than elsewhere, which could again reflect the preference for single-fraction over multiple-fraction radiotherapy.

Our study had some limitations. As discussed in Hoefeler et al., the duration of follow-up for this study was shorter than planned (6.9–10.9 months) owing to slow recruitment (possibly because the trial was non-interventional) and early withdrawal from the study as a result of patient death (Hechmati et al. 2011). The sample sizes for surgery to bone and spinal cord compression were limited and may not have been sufficient to provide a generalizable HRU estimation. Similarly, the applicability of estimations of the duration of inpatient stays may have been limited by the small sample sizes. Other limitations to the study included some data being inaccessible to investigators at all study sites. For example, information about home health visits or nursing home stays was not always relayed back to the main hospital, making it difficult to capture resource use associated with these events. It should also be noted that the incidence of SREs reported here is not representative of the real-world distribution of SRE types, because they are impacted by the index SRE recruitment cells and the inclusion criteria stating patients must have an ECOG performance status of 2 or less and a life expectancy of at least 6 months. Furthermore, pain was not defined as an SRE, although this common complication from poorly treated bone metastases could have led to substantial HRU and lengthy inpatient stays. These limitations would be expected to result in an underestimation of the overall HRU associated with SREs, and therefore the true burden of SREs may be even greater than suggested by these results.

These data indicate that preventing SREs in patients with bone metastases secondary to breast cancer would substantially reduce HRU. Bisphosphonates, such as zoledronic acid, have been widely used to reduce the occurrence of SREs. Denosumab, a monoclonal antibody to RANKL, has recently been approved for the prevention of SREs in patients with bone metastases from solid tumors (European Medicines Agency 2012), and has been shown to be superior to zoledronic acid at reducing the incidence of SREs (Fizazi et al. 2011; Henry et al. 2011; Stopeck et al. 2010).

In conclusion, all types of SRE arising from bone metastases in patients with breast cancer are associated with considerable HRU. Therefore, treatments that delay or prevent SREs may reduce the burden imposed on healthcare systems across Europe.

### Ethical standards

This study was carried out in accordance with the current laws of the countries in which it was performed. Written informed consent was obtained from each patient or their legally acceptable representative. A copy of the protocol, proposed informed consent form, other written patient information, and any proposed advertising material were submitted to the independent ethics committee/institutional review board for written approval where necessary.
